# Salt tolerance and regulation of Na^+^, K^+^, and proline contents in different wild turfgrasses under salt stress

**DOI:** 10.5511/plantbiotechnology.23.0721a

**Published:** 2023-12-25

**Authors:** Yuichi Tada, Ryuto Kochiya, Masayuki Toyoizumi, Yuka Takano

**Affiliations:** 1 School of Bioscience and Biotechnology, Tokyo University of Technology, 1404-1 Katakura, Hachioji, Tokyo 192-0982, Japan

**Keywords:** K^+^, Na^+^, proline, salt tolerance, turfgrass

## Abstract

Turfgrasses show a wide range of salinity tolerance. In this study, twenty wild turfgrasses were collected from coastal regions in Japan, and their species; evolutionary lineage; salt tolerance levels; shoot and root K^+^, Na^+^, and proline contents; and amounts of ions secreted from their salt glands were determined. Among them, eighteen turfgrass species were determined based on the internal transcribed spacer 1 sequences. All collected wild turfgrasses were identified as halophytes and were divided into two salt-tolerant levels. They maintained the shoot relative water contents and suppressed excess Na^+^ accumulation in their shoots and roots and K^+^ content homeostasis compared with rice, resulting in the maintenance of a higher K^+^/Na^+^ ratio under salt stress. These characteristics must be part of the salt tolerance mechanisms. Among the four turfgrasses with salt glands, three selectively secreted Na^+^ from their salt glands; however, interestingly, one secreted K^+^ over Na^+^, although it still maintained a K^+^/Na^+^ ratio comparable to that of the other turfgrasses. A significant amount of proline synthesis was observed in most of the turfgrasses in response to salt stress, and the proline content was highly correlated with the salt tolerance, suggesting its key role in the salt tolerance mechanisms. These wild turfgrasses with such diverse ion control mechanisms and proline synthesis profiles are useful materials for investigating the salt tolerant mechanisms and breeding salt tolerant turfgrasses.

## Introduction

Turfgrass species are in the family *Poaceae*, which was formerly known as *Gramineae* under the class Monocotyledonea. Turfgrasses are widely distributed in various environments across the world and show a wide range of salinity tolerance (Uddin and Juraimi 2013). The demand for salt-tolerant turfgrasses is increasing due to the augmented use of effluent or low-quality water (sea water) for turf irrigation (Uddin and Juraimi 2013; Wang et al. 2020). Wild turfgrasses are also dominant components of communities in coastal dunes and play a fundamental role in stabilizing sand (Balestri and Lardicci 2013). It has been reported that salt-tolerant turfgrasses include alkaligrass (*Puccinellia* spp.), Seashore paspalum (*Paspalum vaginatum*), St. Augustine (*Stenotaphrum secundatum*), and Japanese lawn grass (*Zoysia japonica*) (Marcum 1999; Uddin and Juraimi 2013). We also reported that *Sporobolus virginicus* showed a salinity tolerance of up to 1.5 M NaCl, a three-fold higher concentration than seawater salinity (Tada et al. 2014). Creeping bentgrass (*Agrostis palustris* Huds.) is considered to be relatively salt-tolerant by most investigators, being classified as tolerant of soil ECe from 8 to 16 dS m^−1^ (Marcum 1999). There appears to be a broad range of salinity tolerance within Fescues (*Festuca* spp.) (Marcum 1999). Perennial Ryegrass (*Lolium perenne* L.) is typically ranked as having medium salinity tolerance, tolerating an ECe of 4–8 dS m^−1^, and Kentucky bluegrass (*Poa pratensis* L.) has been ranked as having poor salinity tolerance, tolerating an ECe of less than 4 dS m^−1^ (Marcum 1999). Bermudagrass (*Cynodon* spp.) is a popular and extensively used turf species, and it has a good salt tolerance level with wide intraspecies variations (Dudeck et al. 1983; Shao et al. 2021). *Zoysia* spp. are recognized as excellent warm-season turfgrasses worldwide, with a high salt tolerance and superior growth in saline–alkali soils (Ge et al. 2006; Ming et al. 2022; Qian et al. 2000; Wang et al. 2020; Weng and Chen 2001). All *Zoysia* spp. have salt glands on their leaves, which regulate ion balance by selectively secreting salt ions (Wang et al. 2020). There are also reports comparing the salt tolerance of wild turfgrasses in specific countries or regions (Marcum et al. 2005; Uddin et al. 2011; Weng and Chen 2001).

However, there are no studies comparing salt tolerance of wild turfgrasses with physiological responses such as ion accumulation and biosynthesis of compatible solutes. In this study, we collected wild turfgrasses around the coast of Japan, analyzed their phylogenic relationship, and examined the variations in their salinity tolerance, water contents, Na^+^ and K^+^ contents, and proline contents. The capacity of plants to maintain a high cytosolic K^+^/Na^+^ ratio is likely to be one of the key determinants of plant salt tolerance (Maathuis and Amtmann 1999). Proline has been recognized as a multi-functional molecule, accumulating in high concentrations in response to a variety of abiotic stresses. It is able to protect cells from damage by acting as both an osmotic agent and a radical scavenger (Kavi Kishor and Sreenivasulu 2014; Per et al. 2017; Szabados and Savouré 2010). To the best of our knowledge, only a few reports to date have investigated the proline content of turfgrasses under stress conditions (Li et al. 2018; Long et al. 2020; Marcum and Murdoch 1994; Tada et al. 2014). The wild turfgrasses used in this study exhibited controlled Na^+^ and K^+^ contents and high proline accumulation under salt stress, resulting in high salt tolerance. They are useful materials for investigating the regulation of Na^+^ and K^+^ contents and proline synthesis under salinity and the breeding of salt tolerant turfgrasses.

## Materials and methods

### Plant materials

**Figure 1:**
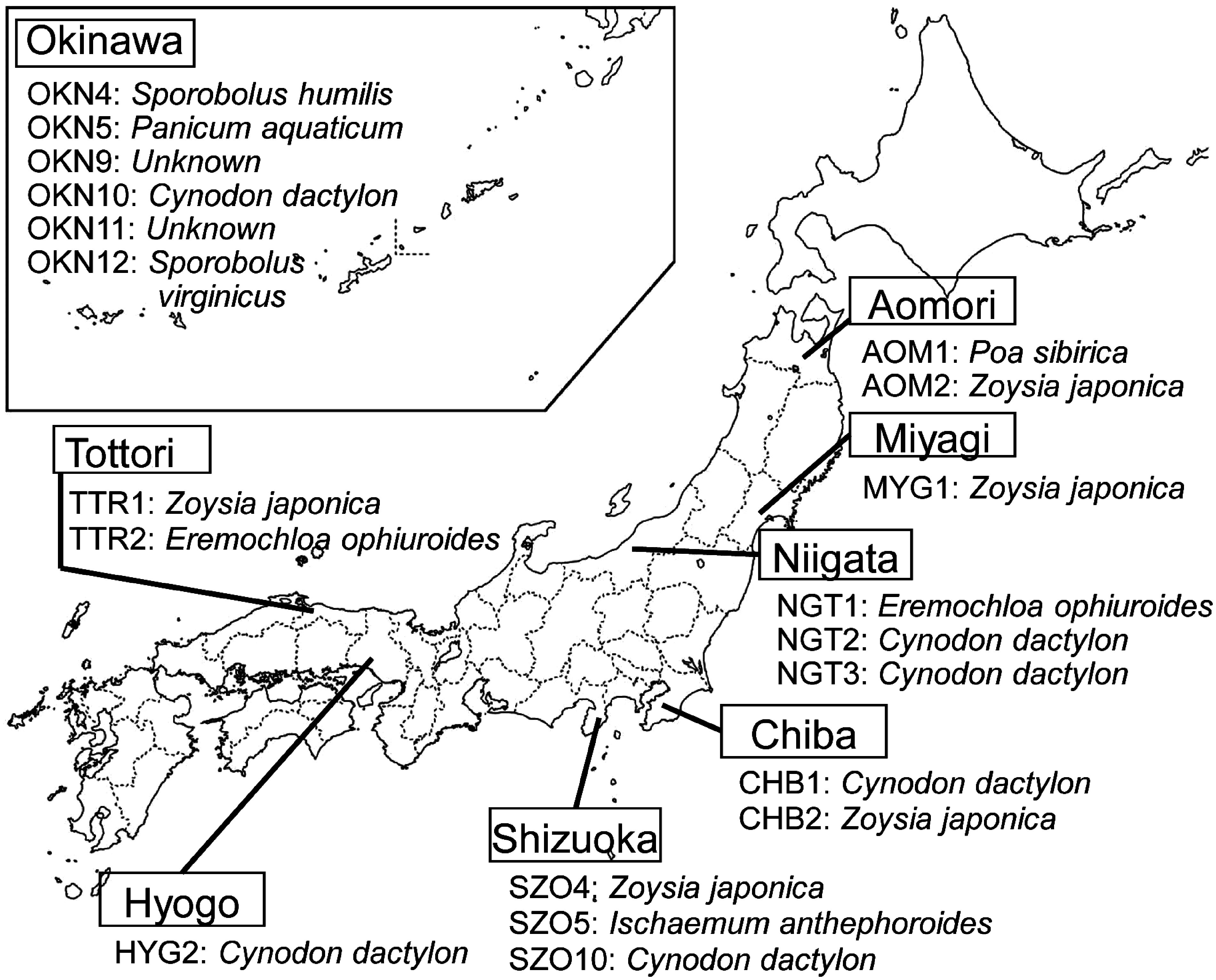
Figure 1. Collection sites (prefectures in Japan) and species identification of wild turfgrasses used in this study. The boxed names are the prefecture names where the plants were collected. Under the prefecture name, provisional plant name(s) based on the prefecture name is shown, followed by the species name identified from the analysis of the ITS1 nucleotide sequence.

We previously collected the rhizomes of wild turfgrass species *Sporobolus virginicus* (OKN12 in this study); OKN4, 5, 9, 10, and 11 from Okinawa prefecture; and NGT1, NGT2, and 3 from Niigata prefecture (Endo et al. 2017; Tada et al. 2014). In this study, we additionally used the rhizomes of wild turfgrasses named TTR1 and TTR2 from Tottori; HYG2 from Hyogo; SZO4, SZO5, and SZO10 from Shizuoka; CHB1 and CHB2 from Chiba; MYG1 from Miyagi; and AOM1 and AOM2 from Aomori prefectures. Their sampling locations are shown on the map in Figure 1. The seeds of crop, turf, and forage plants used in the previous study (Endo et al. 2017), namely, rice (*Oryza sativa* cv. Nipponbare), corn (*Zea mays* cv. Peter corn), Kentucky bluegrass (*Poa pratensis* cv. Moonlight SLT), bent grass (*Agrostis tenuis* Sibth), Italian ryegrass (*Lolium multiflorum* L.), *Brachypodium distachyon* (stiff brome), and perennial ryegrass (*Lolium perenne* L.) and rhizomes of manila grass (*Zoysia matrella* (L.) Merr.) were also used for comparison in this study.

### Species identification and phylogenetic analysis

The internal transcribed spacer 1 (ITS1) sequences between the 18S rRNA and 5.8S rRNA genes were amplified via PCR using specific primers according to the previous report (Endo et al. 2017). The homology of the ITS1 sequences was searched for using blastn search, and the species showing 98% or more homology was identified as the corresponding species. A phylogenetic analysis of the ITS1 sequences was previously performed using the neighbor-joining method and the MEGA-X software package (Kumar et al. 2018), following their alignment using ClustalW.

### Plant growth and salt stress treatments

Turfgrass stolons were cut and placed into culture pots filled with ‘Akadama’ soil (red clay ball) and cultivated for 4 weeks. For comparison, 4-week-old crop, turf, and forage plants including rice were used. The plants were grown in an incubator at 25°C, 70% humidity, and a 12 h light/12 h dark cycle with a photon-flux density of 200 µmol photons m^−2^ s^−1^ under white florescent lights. Propagation of the wild turfgrasses was done by cuttings, as these plants have not flowered for at least 5 years under the conditions.

For the salt tolerance test, the potted plants (3–5 biological replicates) were supplied with a 500 mM NaCl solution through the bottom of their pots. Then, the survival period of these plants was examined and evaluated as follows: level 1 (sensitive), dead within 2 weeks, level 2 (tolerant), dead within 8 weeks, and level 3 (highly tolerant), survived for more than 8 weeks. Plants were judged dead when they lost their green color and did not recover even after being watered.

For the hydroponic culture of the wild turfgrasses, their stolons were cut, rooted, and cultivated in a Hyponica culture solution (Kyowa Co., LTD., Osaka, Japan), which contains 80 mg l^−1^ N, 76 mg l^−1^ P, 188 mg l^−1^ K, and minor elements, in the hydroponic system “Home Hyponica 303” (Kyowa Co., LTD.). To determine the relative water content and the ion contents in the turfgrasses under salt stress, 300 mM NaCl was added to the hydroponic solution; the shoots and roots were sampled at day 0, 1, 3, and 7 after the NaCl treatment; and their fresh weight (FW) and dry weight (DW) were determined. To determine the proline contents, the shoots and roots were collected from the hydroponically cultured plants at day 0 and 7 after the 300 mM NaCl treatment, and their FW was determined. The relative water content of shoot and root was calculated as follows: relative water content (%)=100×(FW−DW)/FW. Three biological replicates were used for all treatments and time points.

### Measurement of ion and proline contents

The contents of Na^+^ and K^+^ in the plants and the amount of secreted Na^+^ and K^+^ from the leaves were determined using an ion analyzer IA-300 (TOA DKK, Tokyo, Japan) according to our previous report (Tada et al. 2014). To collect the salts secreted from the salt glands of the turfgrass leaves, the turfgrass shoots were placed in a centrifuge tube containing deionized water and shaken thoroughly. The concentration of dissolved ions in the water was measured. The ion concentrations are expressed as micromoles per gram DW (µmol g^−1^ DW). The proline contents in the shoots and roots were determined according to the method of Bates et al. (1973) and are expressed as micromoles per gram FW (µmol g^−1^ FW).

### Data analysis

The experiment was performed with at least three biological replications per treatment (*n*=3–5). All experimental results were presented as the mean±standard error. Mean comparison was performed using Tukey’s multiple contrasts method.

## Results

### Collection and characterization of wild turfgrasses

We collected 20 wild turfgrasses from the coastal regions of eight prefectures in Japan (Figure 1). These wild turfgrasses were given a tentative name by combining the abbreviation of the “Prefecture name” where they were collected and the number of the order in which they were collected; the plants collected in Okinawa prefecture were called OKN1, OKN2, and so on. To identify the species of the collected wild turfgrasses, the nucleotide sequences of their ITS1 sequences were sequenced and searched for on the blastn database. If the sequences showed 98% or more homology to the sequences of registered plant species, they were judged to be the same species. Out of the 20 wild turfgrasses, we were able to identify the species of 18 turfgrasses, but 2 turfgrasses, OKN9 and OKN11 showed 91% and 96% homology with *Urochloa dictyoneura* and *Paspalum scrobiculatum*, respectively, and were judged to be ‘unknown’ (Figure 1).

**Figure 2:**
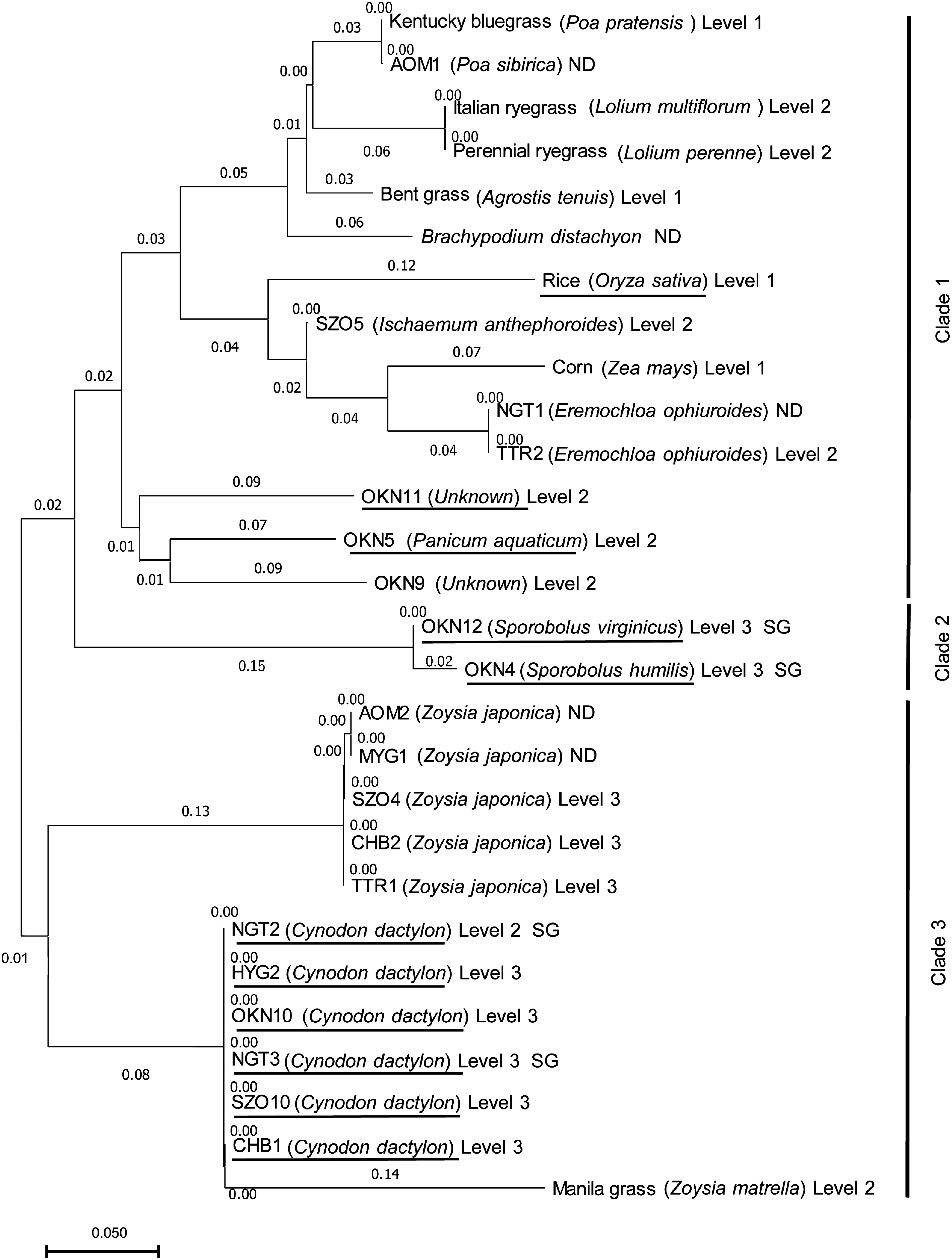
Figure 2. Phylogenetic analysis and salt tolerance of wild turfgrasses. Phylogenetic analysis was performed based on the ITS1 nucleotide sequences of twenty wild turfgrasses and eight commercially available plants using the neighbor-joining method. The evolutionary distances were computed using the Maximum Composite Likelihood method and are in the units of the number of base substitutions per site. The scale bar shows a length corresponding to 0.05 of the value. Salt tolerance of plants was evaluated as follows based on the survival period when cultivated under 500 mM NaCl. Level 1, died within 2 weeks; Level 2, died within 8 weeks; Level 3, did not die longer than 8 weeks. ND, salt tolerance was not determined. SG indicates a plant with visible salt secretion from salt glands under salt stress. Underlined plants were subjected to quantification of ions and proline contents.

A phylogenetic analysis of the wild turfgrasses was performed based on the ITS1 sequences (Figure 2). The plants were roughly divided into three clades via the phylogenetic analysis: clade 1, which included rice and *Poa*, *Eremochloa*, *Ischaemum*, and *Panicum* genera, clade 2, which included *Sporobolus* genus, and clade 3, which included *Cynodon* and *Zoysia* genera.

Then, we examined the salinity tolerance levels of the selected 16 wild turfgrasses and 8 commercially available crop, turf, and forage plants. After treatment with 500 mM NaCl, the rice, corn, Kentucky bluegrass (*P. pratensis*), and bent grass (*A. tenuis*) died within 2 weeks (salt tolerance was marked as level 1). Six wild turfgrasses, OKN5 (*Panicum aquaticum*), OKN9 (unknown), OKN11 (unknown), SZO5 (*Ischaemum anthephoroides*), TTR2 (*E. ophiuroides*), and NGT2 (*C. dactylon*) and three commercially available plants, Italian ryegrass (*L. multiflorum*), perennial ryegrass (*L. perenne*), and manila grass (*Z. matrella*), died within 4 weeks (level 2) after salt treatment, but the remaining 10 wild turfgrasses did not die, even after 8 weeks of salt treatment (level 3) (Figure 2, Supplementary Figure S1). All tested wild turfgrasses belonging to clade 1 showed level 2 salt tolerance, but they could survive under 300 mM NaCl (data not shown). The wild turfgrasses belonging to clades 2 and 3 showed level 3 salt tolerance, except for NGT2 (*C. dactylon*). Four wild turfgrasses, OKN4 (*Sporobolus humilis*), OKN12 (*S. virginicus*), NGT2 (*C. dactylon*), and NGT3 (*C. dactylon*), were found to have salt glands (SGs) because salt crystals were observed on their leaf surfaces when they were under salt stress (Figure 2). They were divided into the two clades (clades 2 and 3). The secretion of salt was not observed on the leaves of the examined *Z. japonica* (TTR1, CHB2, and SZO4), although it has been reported that all Zoysia spp. have salt glands on their leaves (Wang et al. 2020).

### Relative water content in wild turfgrasses

**Figure 3:**
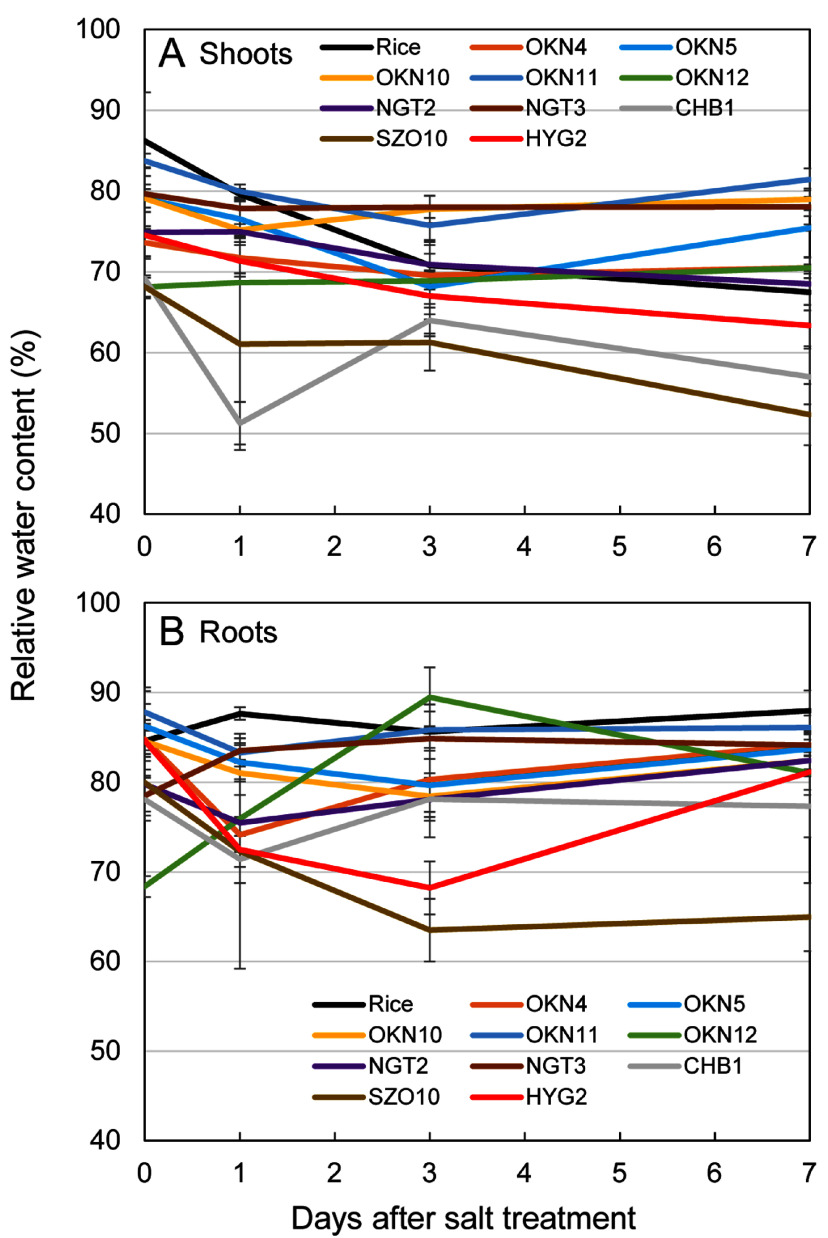
Figure 3. Changes of relative water content of wild turfgrasses and rice under 300 mM NaCl condition. The relative water content in shoot (A) and root (B) at day 0, 1, 3, and 7 after salt treatment. See Supplementary Table S1 for statistical analysis of each mean for relative water content.

To evaluate the ability of the wild turfgrasses to maintain the water content under salt stress, we measured the changes in the relative water contents in 10 wild turfgrasses: the most widely distributed turfgrass species, *C. dactylon* (namely, OKN10, HYG2, NGT2, NGT3, SZO10, and CHB1), and the turfgrasses collected from Okinawa prefecture, where the turfgrass species are the most genetically diverse, namely, OKN4 (*S. humilis*), OKN5 (*P. aquaticum*), OKN10 (*C. dactylon*), OKN11 (unknown), and OKN12 (*S. virginicus*). Rice was also used for comparison. The relative water contents in their shoot and root were determined at day 0, 1, 3, and 7 after treatment with 300 mM NaCl (Figure 3, Supplementary Figure S2, Supplementary Table S1). The shoot relative water contents of the wild turfgrasses, except for SZO10 (*C. dactylon*), after salt treatment were similar to those before salt treatment (Figure 3A, Supplementary Table S1). On the other hand, the relative water content in rice shoot was significantly decreased by salt treatment. The root relative water contents in most grasses and rice were unchanged by salt treatment, but it was significantly increased by salt treatment in OKN12 (*S. virginicus*), indicating the unique ability of OKN12 to adapt to salt stress.

### Na^+^ and K^+^ contents in wild turfgrasses

**Figure 4:**
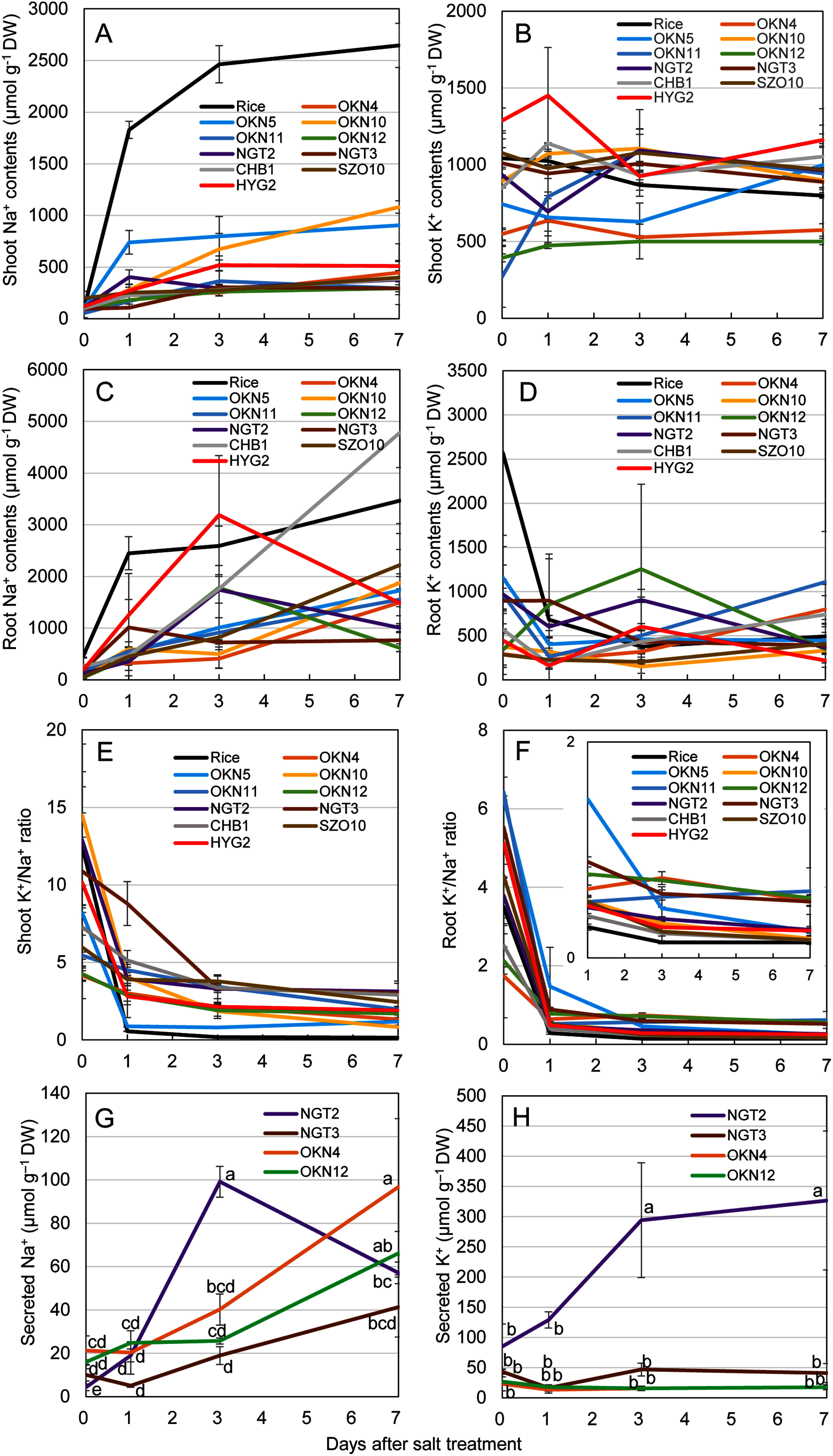
Figure 4. Changes of Na^+^ and K^+^ contents, K^+^/Na^+^ ratio, and secreted Na^+^ and K^+^ of wild turfgrasses and rice under 300 mM NaCl condition. Changes in shoot Na^+^ (A) and K^+^ (B), root Na^+^ (C) and K^+^ (D) contents, the shoot K^+^/Na^+^ (E) and root K^+^/Na^+^ ratios (F), and amounts of secreted Na^+^ (G) and K^+^ (H) at day 0, 1, 3, and 7 after salt treatment. Means of secreted ions with different letters are significantly different at *p*<0.05 using Tukey’s method. See Supplementary Table S2 for statistical analysis of each mean for Na^+^ and K^+^ content and K^+^/Na^+^ ratio.

To understand the ion management of the wild turfgrasses under salt stress, we determined the changes in Na^+^ and K^+^ contents in the shoots and roots of the wild turfgrasses at day 0, 1, 3, and 7 after treatment with 300 mM NaCl (Figure 4, Supplementary Figure S2, and Supplementary Table S2). The shoot Na^+^ content in the turfgrasses rose moderately after 1–3 days of salt treatment but almost plateaued after that. However, the shoot Na^+^ content of the rice increased sharply one day after salt treatment. The root Na^+^ content after salt treatment was lower in the wild turfgrasses than in the rice, except for that of CHB1 (*C. dactylon*). The root Na^+^ content in CHB1 (*C. dactylon*) increased with time and exceeded that in rice roots at day 7 . The change patterns of the root Na^+^ content in the other turfgrasses were divided into two types. One is a moderately increasing type, as observed in OKN4 (*S. humilis*), OKN5 (*P. aquaticum*), OKN10 (*C. dactylon*), OKN11 (unknown), and SZO10 (*C. dactylon*), and the other is a type that peaks on day 1 or 3 and then shows decreasing, as observed in OKN12 (*S. virginicus*), NGT2 (*C. dactylon*), NGT3 (*C. dactylon*), and HYG2 (*C. dactylon*). Thus, the changes in the Na^+^ content in the wild turfgrasses under salt stress were tightly regulated to prevent rapid increases and excessive accumulation, except for that in the roots of CHB1 (*C. dactylon*).

Shoot K^+^ content under non-stress conditions varied greatly among the wild turfgrass, from 270 µmol g^−1^ DW in OKN11 (unknown) to 1,288 µmol g^−1^ DW in HYG2 (*C. dactylon*) (Figure 4B, Supplementary Table S2). This was 1,042 µmol g^−1^ DW in the rice. The shoot K^+^ content in the rice decreased by more than 20% at day 7 after treatment with 300 mM NaCl. In contrast, the contents in the turfgrasses increased or unchanged in 7 lines and slightly decreased in 3 lines. However, the root K^+^ content under non-stress conditions was the highest (2,570 µmol g^−1^ DW) in the rice and the lowest (286–1,156 µmol g^−1^ DW) in the turfgrass. After salt treatment, a rapid decrease in root K^+^ content was observed in the rice (about 74 and 81% decreases at day 1 and 7, respectively), but it decreased by 56–62% after 7 days in OKN5 (*P. aquaticum*), NGT2 (*C. dactylon*), NGT3 (*C. dactylon*), and HYG2 (*C. dactylon*); did not change significantly in OKN10 (*C. dactylon*) and OKN12 (*S. virginicus*); and instead increased in OKN4 (*S. humilis*) (2.71 fold), OKN11 (unknown) (1.15 fold), CHB1 (*C. dactylon*) (1.30 fold), and SZO10 (*C. dactylon*) (1.44 fold). Thus, the wild turfgrasses had a better ability to maintain K^+^ content homeostasis than the rice, or they have a mechanism to enhance K^+^ absorption and accumulation under salt stress. As a result, the K^+^/Na^+^ ratios in the shoots and roots of the wild turfgrasses were kept relatively higher than that of the rice under salt stress (Figure 4E, F).

Because four wild turfgrasses, OKN4 (*S. humilis*), OKN12 (*S. virginicus*), NGT2 (*C. dactylon*), and NGT3 (*C. dactylon*), formed salt crystals on their leaves after salt treatment, we measured the amounts of secreted Na^+^ and K^+^ (Figure 4G). The secretion of Na^+^ increased in OKN4 (*S. humilis*), OKN12 (*S. virginicus*), and NGT3 (*C. dactylon*) on the 7th day after salt treatment but peaked at day 3 in NGT2 (*C. dactylon*). Interestingly and surprisingly, the secretion of K^+^ increased dramatically in NGT2 but remained unchanged in the other three after salt treatment (Figure 4H). As a result, OKN4 (*S. humilis*), OKN12 (*S. virginicus*), and NGT3 (*C. dactylon*) selectively secreted Na^+^, but NGT2 (*C. dactylon*) secreted K^+^ over Na^+^.

### Proline contents in wild turfgrasses

**Figure 5:**
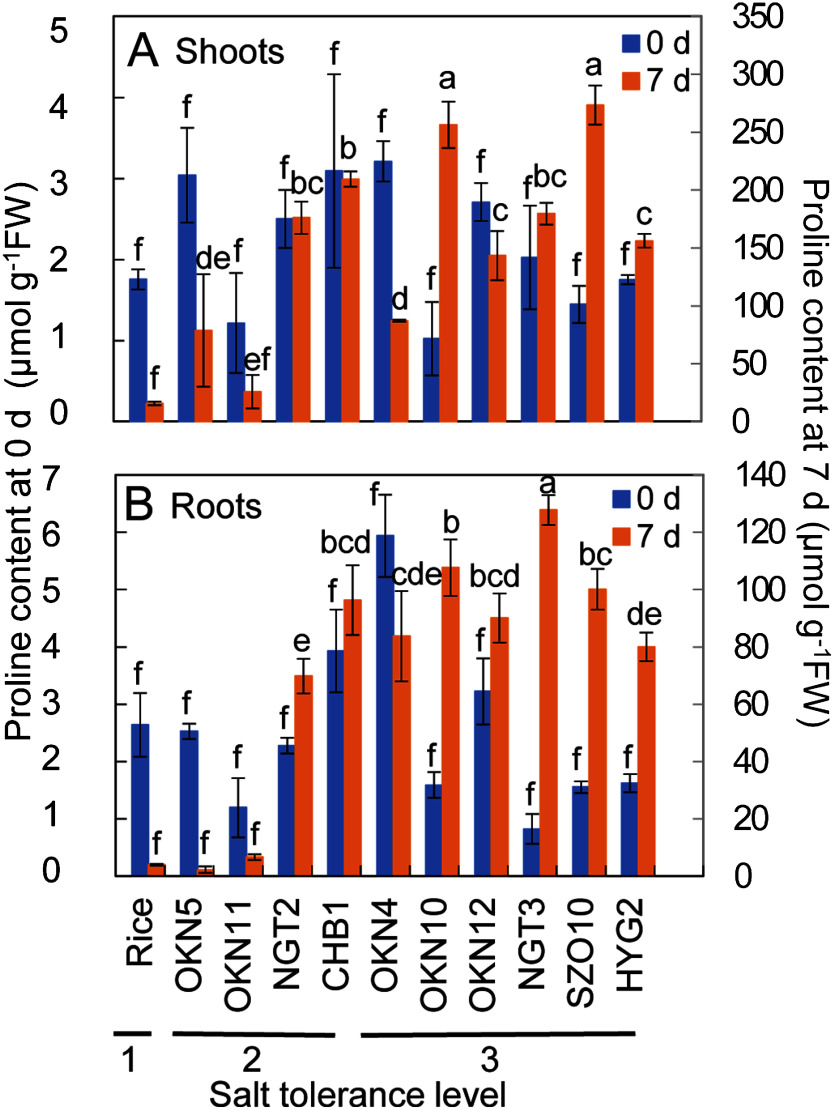
Figure 5. Changes of proline contents of wild turfgrasses and rice under 300 mM NaCl condition. The proline contents were determined in shoots and roots of turfgrasses and rice hydroponically cultivated with 300 mM NaCl for 0 and 7 day. (A) Shoot proline content. (B) Root proline content. Means with different letters are significantly different at *p*<0.05 using Tukey’s method.

We examined the changes in the proline contents in the wild turfgrasses and the rice treated with 300 mM NaCl because proline has been reported to play an important role in salt tolerance of some turfgrasses (Li et al. 2018; Long et al. 2020; Marcum and Murdoch 1994; Tada et al. 2014). The proline contents in the shoots and roots were determined at day 0 and 7 after salt treatment (Figure 5 and Supplementary Table S3). Under the non-stressed condition (at day 0), the proline contents in the shoots and roots of the wild turf grasses and the rice were very small and negligible amount as an osmolyte (almost 1–6 µmol g^−1^ FW). After salt treatment, the shoot proline content in the turfgrasses increased dramatically and was the highest in SZO10 (*C. dactylon*) (273 µmol g^−1^ FW). The maximum increase rate was observed in OKN10 (*C. dactylon*) (251-fold increase). The shoot proline content in OKN11 (unknown), which was the least abundant in the turfgrasses under salt stress, was 25.8 µmol g^−1^ FW. The shoot proline content in the rice was only 15.7 µmol g^−1^ FW, even after salt treatment. The root proline content in the salt-treated turfgrasses was lower than that in the shoots, up to 128 µmol g^−1^ FW in NGT3 (*C. dactylon*). The content in the non-stressed rice roots was only 4 µmol g^−1^ FW and increased by 1.5-fold after salt treatment. Among the wild turfgrasses, the root proline contents in OKN5 (*P. aquaticum*) and OKN11 (unknown) were 2.2 and 6.7 µmol g^−1^ FW, respectively, which were as low as that in the rice. It is worth noting that OKN5 (*P. aquaticum*) and OKN11 (unknown) were classified as having level 2 salt tolerance (Figure 2). The root proline contents in the other turfgrasses reached more than 60 µmol g^−1^ FW after salt stress.

## Discussion

We collected 20 wild turfgrasses in Japan and identified the species of 18 plants based on their ITS1 sequences (Figure 1). Because the turfgrasses survived under 300 mM NaCl, all wild turfgrasses used were categorized as halophytes. Among them, OKN4 (*S. humilis*), OKN12 (*S. virginicus*), NGT2 (*C. dactylon*), and NGT3 (*C. dactylon*) had salt glands. Salt glands are highly evolved structures with the main function of secreting excessive salt outside the body to avoid high levels of accumulation in the cells (Flowers and Colmer 2008). These four turfgrasses belonged to different species, were divided into different clades via the phylogenetic analysis, and showed different levels of salt tolerance. Halophytes are widely but unevenly spread over higher plant families (Flowers and Colmer 2008). The *Poales* order contains ∼8% of all halophytes (Flowers et al. 2010) and has been the focus of many salt-gland-focused studies (Céccoli et al. 2015). Adaptation to high salt concentrations in higher plants does not appear to be a relic ability inherited from marine ancestors, but it appears to have evolved repeatedly (>70 times in grass lineages) and independently in a wide range of taxa (Bennett et al. 2013; Flowers et al. 2010; Maas 1986). Our results also suggest that the acquisition of salt glands during the evolution of the wild turfgrasses are independent and polygenic.

Maintaining a high cytosolic K^+^ concentration or a high K^+^/Na^+^ ratio is a key factor of salt tolerance mechanisms and depends on the characteristics of the high-affinity K^+^ transporters (HKTs) that mediate K^+^ and Na^+^ absorption (Maathuis and Amtmann 1999; Rubio et al. 2020; Shabala and Cuin 2008). Differences in the regulation and the function of these transporters among species may lead to different controls of K^+^/Na^+^ homeostasis that result in different salt tolerance levels (Alemán et al. 2009). All wild turfgrasses used in this study suppressed excess Na^+^ accumulation in their shoots and roots and maintained K^+^ content homeostasis, resulting in the maintenance of a higher K^+^/Na^+^ ratio under salt stress (Figure 4). These turfgrasses may be excellent materials for analyzing the function of transporters regulating K^+^ and Na^+^ absorption and transport in halophytes.

*Cynodon* spp. and *Zoysia* spp. have been reported to show high salinity tolerance levels (Dudeck et al. 1983; Marcum et al. 1998; Shao et al. 2021; Weng and Chen 2001). In this study, among six *C. dactylon*, five (OKN10, HYG2, NGT3, CHB1, and SZO10) showed high salt tolerance (level 3) and one (NGT2) showed moderate salt tolerance (level 2) (Figure 2). Both NGT2 and NGT3 belonged to the same species (*C. dactylon*), accumulated comparable amounts of proline under 300 mM NaCl (Figure 5), and had salt glands; however, NGT2 secreted an extremely high amount of K^+^ from its salt glands under salinity compared with the other three turfgrasses with salt glands (Figure 4H). This may be the reason why the salt tolerance level of NGT2 was inferior to that of the other turfgrasses with salt glands because maintaining a high K^+^ content is a key factor in salt tolerance mechanisms; however, surprisingly, the K^+^ content and the K^+^/Na^+^ rate in NGT2 (*C. dactylon*) were comparable to those of the other turfgrasses despite its abundant K^+^ secretion (Figure 4H). The relative salinity tolerance in the *Zoysia* genus has been found to be positively correlated with the salt gland Na^+^ secretion rate and leaf gland density (Marcum et al. 1998). NGT3 (*C. dactylon*), OKN4 (S. *humilis*), and OKN12 (*S. virginicus*) in this study and *S. virginicus* (OKN12) in our previous study (Tada et al. 2014) were found to selectively secrete Na^+^ from their salt glands. The selective secretion of K^+^ over Na^+^ observed in NGT2 (*C. dactylon*) (Figure 4G, H) is very rare and contrary to the maintenance of K^+^ content, a known mechanism of salt tolerance. NGT2 (*C. dactylon*) may be able to maintain its K^+^ content by its high K^+^ absorption ability even though it secretes more K^+^ than other turfgrasses. More detailed research may be needed to uncover the role of salt glands in salt tolerance mechanisms in this turfgrass.

Proline is able to protect cells from damage by acting as both an osmotic agent and a radical scavenger, and proline accumulation is one of the striking metabolic responses of plants to salt stress (Kavi Kishor and Sreenivasulu 2014; Per et al. 2017; Szabados and Savouré 2010). The proline contents in salt-treated shoots were higher in bermudagrass (*C. dactylon*) (34 mM) than in manila grass (*Z. matrella*), Seashore paspalum, St. Augustine, centepedegrass, and Japanese lawn grass (*Z. japonica*) (Marcum and Murdoch 1994). The proline contents in two turfgrass genotypes of *Carex rigescens* were about 100 µg g^−1^ FW (0.87 µmol g^−1^ FW) under the non-stress condition and increased to 400 or 550 µg g^−1^ FW (3.47–4.78 µmol g^−1^ FW) under the 300 mM NaCl condition (Li et al. 2018). We also reported the salt responsive accumulation of proline (Tada et al. 2014) and the transcriptional activation of a gene for pyrroline-5-carboxylate synthetase (P5CS) (Yamamoto et al. 2015), which catalyze the rate-limiting step of glutamate-derived proline biosynthesis in OKN12 (*S. virginicus*). In this study, the proline contents in the shoots and roots of the turfgrasses, except for in those of OKN5 (*P. aquaticum*) and OKN11 (unknown), increased to 87–273 µmol g^−1^ FW and 70–128 µmol g^−1^ FW, respectively, under 300 mM NaCl, from negligible amounts (1–6 µmol g^−1^ FW) under the non-stress condition (Figure 5). These elevated proline contents have the effect of significantly increasing the intracellular osmotic pressure against osmotic stress caused by salt stress. Therefore, proline synthesis is considered to play an important role as one of the salt tolerance mechanisms of these wild turfgrasses. Such remarkable proline synthesis responding to salt stress was not observed in rice, OKN5 (*P. aquaticum*), or the roots of OKN11 (unknown), suggesting that it is one of the reasons for the level 2 salt tolerance of these turfgrasses. These wild turfgrasses should be excellent research materials of proline biosynthesis at molecular level under salt stress.

Accumulation of Na^+^ and proline as osmolytes in the wild turfgrasses should contribute to maintain the high shoot water contents (Figure 3) by reducing the water potential. Halophytes accumulate Na^+^ into vacuoles, and this provides the osmotic potential that supports water influx and accelerates growth (Adams et al. 1998; Ahmadi et al. 2022; Tester and Davenport 2003). Besides that, these wild turfgrasses suppressed excess Na^+^ accumulation and maintained K^+^ content homeostasis, resulted in maintaining higher K^+^/Na^+^ ratio under salt stress. Among four turfgrasses having salt grands, one (NGT2) secreted more K^+^ over Na^+^ though NGT2, despite showing a comparable K^+^/Na^+^ ratio to the other turfgrasses. These wild turfgrasses with such diverse ion control mechanisms and proline synthesis profiles are useful materials for investigating the salt tolerant mechanisms and breeding salt tolerant turfgrasses.

## Abbreviations

HKT: high-affinity K^+^ transporter
ITS1: internal transcribed spacer 1
P5CS: pyrroline-5-carboxylate synthetase

## Description of Supplementary Files

Table S1 Changes of relative water content of wild turfgrasses and rice under 300 mM NaCl condition. Table S2 Changes in Na^+^ and K^+^ contents and K^+^/Na^+^ ratio of wild turfgrasses and rice under 300 mM NaCl condition. Table S3 Changes in proline contents in shoots and roots of turfgrasses and rice under 300 mM NaCl condition. Figure S1 Appearance of wild turfgrasses grown on soil treated with 500 mM NaCl for 8 weeks. Figure S2 Appearance of wild turfgrass and rice plants hydroponically grown in 300 mM NaCl for 7 days.
