# Supplementary material for: Salt tolerance and regulation of Na+, K+, and proline contents in different wild turfgrasses under salt stress
Source: Plant Biotechnol (Tokyo). 2023 Dec 25;40(4):301–9. doi: 10.5511/plantbiotechnology.23.0721a (PMC10904837; doi:10.5511/plantbiotechnology.23.0721a)
Supplement: Supplementary Data [file plantbiotechnology-40-4-23.0721a-s001.pdf]

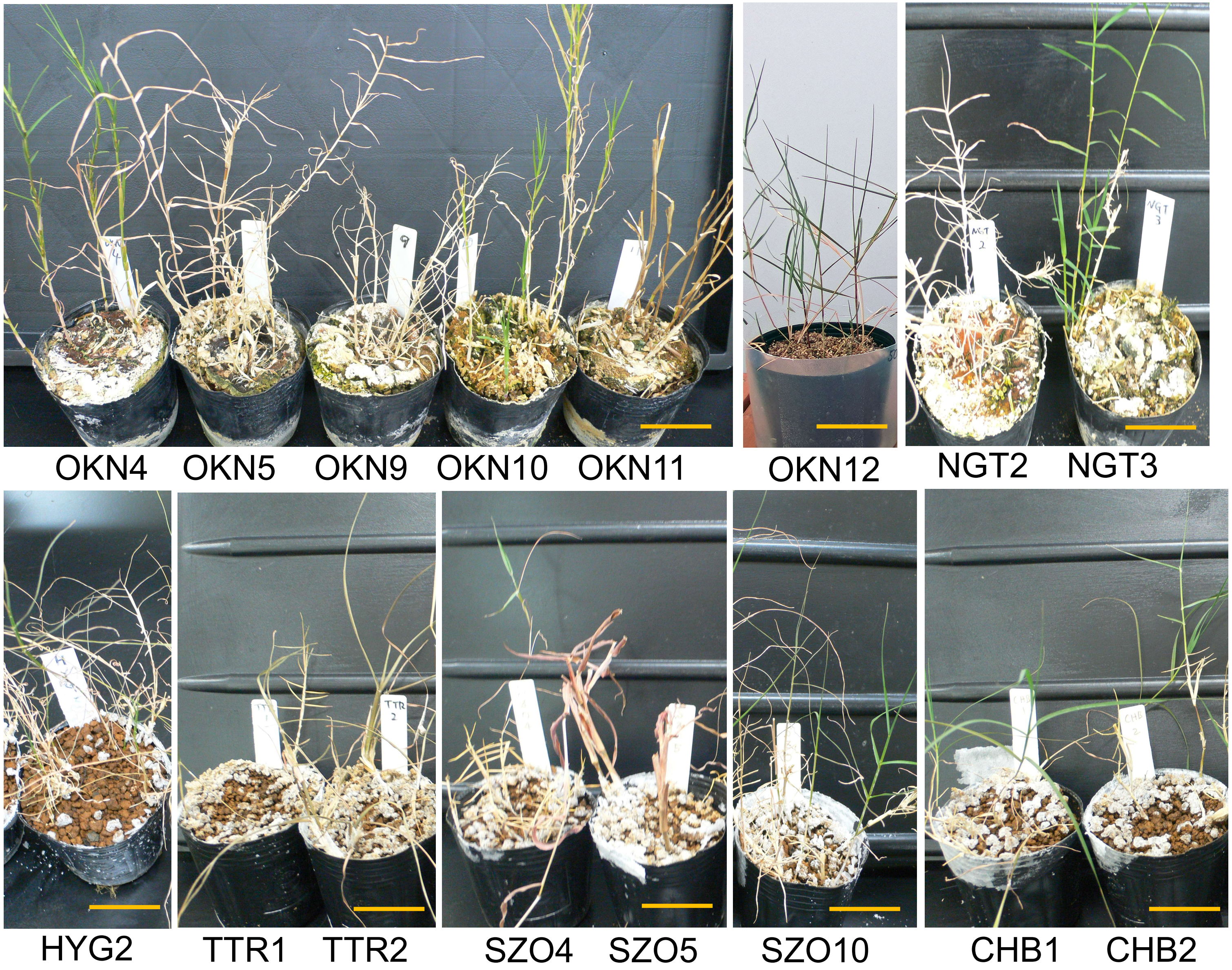

Supplementary Figure S1. Appearance of wild turfgrasses grown on soil treated with 500 mM NaCl for 8 weeks. OKN4, OKN10, OKN12, NGT3, HYG2, TTR1, SZO4, SZO10, CHB1, and CHB2 remained green after the treatment, indicating that they were alive. The other turfgrasses lost their green color and were judged dead because they did not recover even after being watered. Yellow bars indicate 5 cm.

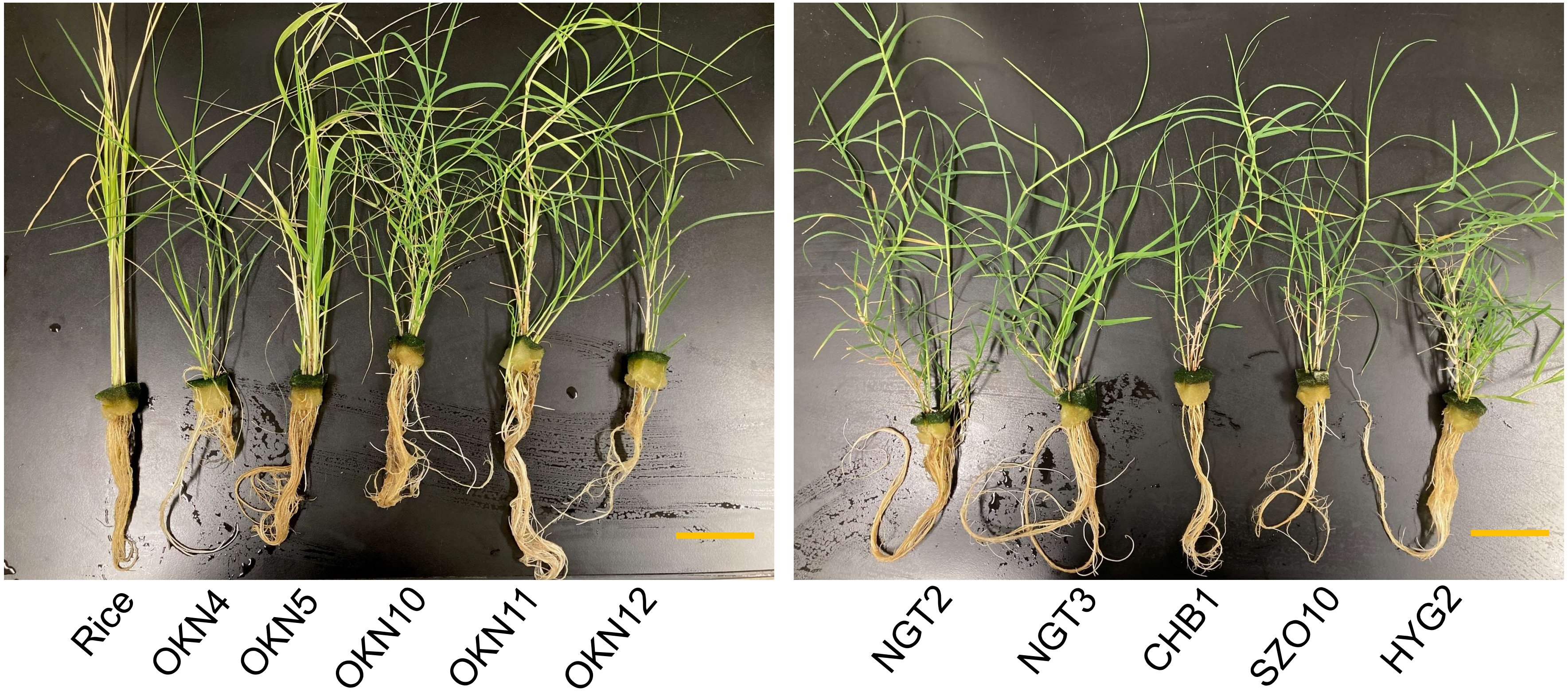

Supplementary Figure S2. Appearance of wild turfgrass and rice plants hydroponically grown in 300 mM NaCl for 7 days. Yellow bars indicate 5 cm.

Supplementary Table S1. Changes in relative water content of wild turfgrasses and rice under 300 mM NaCl condition

A. Shoot relative water content (n=3, biological replications)

Plants	Species	Average shoot relative water content (%)								7d/0d ratio	SE			
		0d	Statistical significance	1d	Statistical significance	3d	Statistical significance	7d	Statistical significance		0d	1d	3d	7d
Rice	*O. sativa*	86.2	a	79.6	abcde	70.8	cdefghijk	67.5	ghijkl	0.78	6.0	0.7	3.0	2.3
OKN4	*S. humilis*	73.6	bcdefghij	71.7	cdefghijk	69.5	defghijk	70.5	defghijk	0.96	1.7	1.9	0.7	1.3
OKN5	*P. aquaticum*	79.1	abcdef	76.5	abcdefgh	68.2	ghijkl	75.5	bcdefghi	0.95	3.9	2.2	5.8	0.4
OKN10	*C. dactylon*	79.1	abcdef	75.2	bcdefghi	77.7	abcdefgh	79.0	abcdef	1.00	1.7	0.7	1.7	1.3
OKN11	*Unknown*	83.7	ab	80.0	abcd	75.7	abcdefghi	81.4	abc	0.97	0.9	0.8	2.0	1.3
OKN12	*S. Virginicus*	68.1	ghijkl	68.7	fghijkl	68.9	fghijkl	70.5	defghijk	1.04	1.2	0.2	3.3	0.4
NGT2	*C. dactylon*	74.9	bcdefghi	74.9	bcdefghi	70.9	cdefghijk	68.5	fghijkl	0.91	3.1	3.1	2.4	3.3
NGT3	*C. dactylon*	79.7	abcde	77.9	abcdef	78.0	abcdefg	78.1	abcdefg	0.98	2.2	1.4	1.4	1.2
CHB1	*C. dactylon*	69.2	efghijkl	51.3	m	64.0	ijkl	57.0	lm	0.82	2.4	2.6	2.0	3.4
SZO10	*C. dactylon*	68.1	ghijkl	61.0	klm	61.3	klm	52.3	m	0.77	1.4	13.1	3.5	3.8
HYG2	*C. dactylon*	74.5	bcdefghi	71.3	cdefghijk	67.0	hijkl	63.4	jkl	0.85	1.1	1.9	3.0	2.6

Means with different letters (Statistical significant) are significantly different at $p < 0.05$ using Tukey's method.

B. Root relative water content (n=3, biological replications)

Plants	Species	Average root relative water content (%)								7d/0d ratio	SE			
		0d	Statistical significance	1d	Statistical significance	3d	Statistical significance	7d	Statistical significance		0d	1d	3d	7d
Rice	*O. sativa*	84.6	abcdef	87.6	abc	85.6	abcde	88.0	ab	1.04	1.1	0.7	0.4	0.6
OKN4	*S. humilis*	84.8	abcdef	74.1	ijklm	80.3	cdefghi	84.2	abcdef	0.99	0.5	1.8	2.8	1.0
OKN5	*P. aquaticum*	86.3	abcd	82.2	abcdefghi	79.6	defghijk	83.7	abcdef	0.97	0.4	1.5	3.5	0.5
OKN10	*C. dactylon*	84.6	abcdef	81.0	bcdefghi	78.4	efghijkl	82.4	abcdefghi	0.97	0.8	0.8	3.1	4.8
OKN11	*Unknown*	87.8	ab	83.3	abcdefg	85.8	abcde	86.1	abcd	0.98	1.2	0.7	0.6	1.1
OKN12	*S. Virginicus*	68.3	mno	75.9	ghijkl	89.4	a	81.0	bcdefghi	1.19	5.0	1.7	4.6	0.5
NGT2	*C. dactylon*	79.8	defghijk	75.5	hijklm	78.1	fghijkl	82.4	abcdefgh	1.03	0.3	1.0	1.6	2.0
NGT3	*C. dactylon*	78.5	efghijkl	83.5	abcdef	84.9	abcdef	84.2	abcdef	1.07	2.8	0.3	0.5	1.7
CHB1	*C. dactylon*	78.1	fghijkl	71.4	lmno	78.1	fghijkl	77.3	fghijkl	0.99	3.6	1.5	2.0	5.4
SZO10	*C. dactylon*	80.0	defghij	72.3	klmn	63.5	o	64.9	no	0.81	0.8	2.4	0.6	3.7
HYG2	*C. dactylon*	84.6	abcdef	72.5	jklm	68.2	mno	81.1	bcdefghi	0.96	0.4	1.4	2.6	1.0

Means with different letters (Statistical significant) are significantly different at $p < 0.05$ using Tukey's method.

Supplementary Table S2. Changes in Na^+ and K^+ contents and K^+/Na^+ ratio of wild turfgrasses and rice under 300 mM NaCl condition

A. Shoot Na^+ content (n=3, biological replications)

Plants	Species	Average shoot Na^+ content (μmol g^{-1} DW)								7d/0d ratio	SE			
		0d	Statistical significance	1d	Statistical significance	3d	Statistical significance	7d	Statistical significance		0d	1d	3d	7d
Rice	*O. sativa*	102.1	lm	1828.4	b	2463.8	a	2644.9	a	25.9	31.5	82.0	179.4	213.5
OKN4	*S. humilis*	134.9	ijklm	226.1	hijklm	253.8	ghijklm	445.7	fgh	3.3	20.3	44.5	30.0	70.3
OKN5	*P. aquaticum*	106.7	klm	739.9	de	799.3	d	904.3	cd	8.5	21.8	114.6	190.5	178.9
OKN10	*C. dactylon*	73.9	m	282.4	ghijklm	673.9	def	1081.2	c	14.6	21.8	48.6	151.7	59.6
OKN11	*Unknown*	55.0	m	170.4	hijklm	363.0	ghijkl	441.3	fgh	8.0	44.2	61.9	99.1	79.9
OKN12	*S. Virginicus*	95.8	lm	180.5	hijklm	262.3	ghijklm	293.5	ghijklm	3.1	17.3	38.3	19.5	9.5
NGT2	*C. dactylon*	109.0	klm	403.6	fghi	294.9	ghijklm	379.7	ghijk	3.5	33.3	68.3	29.1	21.6
NGT3	*C. dactylon*	94.5	lm	107.8	klm	305.8	ghijklm	295.7	ghijklm	3.1	6.9	8.1	27.0	34.1
CHB1	*C. dactylon*	99.1	lm	227.8	hijklm	281.2	ghijklm	384.8	ghij	3.9	29.9	24.4	32.3	71.7
SZO10	*C. dactylon*	197.2	hijklm	253.2	hijklm	274.9	ghijklm	398.9	ghijklm	2.0	68.0	25.0	55.6	165.7
HYG2	*C. dactylon*	119.8	jklm	273.4	ghijklm	517.4	efg	509.4	efgh	4.3	32.0	31.6	51.8	42.9

Means with different letters (Statistical significant) are significantly different at $p < 0.05$ using Tukey's method.

B. Shoot K^+ content (n=3, biological replications)

Plants	Species	Average shoot K^+ content (μmol g^{-1} DW)								7d/0d ratio	SE			
		0d	Statistical significance	1d	Statistical significance	3d	Statistical significance	7d	Statistical significance		0d	1d	3d	7d
Rice	*O. sativa*	1042.2	abcdefg	1025.8	abcdefg	868.4	bcdefghi	799.1	bcdefghij	0.8	69.8	30.5	33.8	18.5
OKN4	*S. humilis*	548.7	efghij	636.3	cdefghij	528.2	fghij	575.6	defghij	1.0	39.1	11.6	9.0	40.5
OKN5	*P. aquaticum*	744.4	bcdefghij	656.8	cdefghij	628.6	cdefghij	998.7	abcdefgh	1.3	239.5	121.0	121.7	113.7
OKN10	*C. dactylon*	891.0	bcdefghi	1071.8	abcdef	1105.1	abcd	898.7	bcdefghi	1.0	42.0	72.5	93.3	47.6
OKN11	*Unknown*	269.8	j	791.9	bcdefghij	1093.2	abcde	943.2	abcdefgh	3.5	198.2	308.7	137.9	106.3
OKN12	*S. Virginicus*	392.7	ij	473.5	hij	499.6	ghij	500.0	ghij	1.3	25.6	20.0	113.0	20.7
NGT2	*C. dactylon*	934.6	ab	694.9	a	1093.6	abcdefghi	962.4	abc	1.0	357.2	127.4	141.6	175.0
NGT3	*C. dactylon*	1009.4	abcdefgh	943.6	abcdefgh	1008.1	abcdefgh	888.0	bcdefghi	0.9	159.1	144.1	13.3	92.5
CHB1	*C. dactylon*	848.1	abc	1139.3	abc	932.1	abcdefghi	1053.4	abcdef	1.2	373.3	4.5	60.4	204.4
SZO10	*C. dactylon*	1076.5	abcde	976.9	abcdefgh	1076.9	abcde	967.5	abcdefgh	0.9	212.0	66.6	282.1	398.0
HYG2	*C. dactylon*	1287.6	cdefghij	1450.4	cdefghij	924.4	abcde	1161.5	abcdefgh	0.9	81.7	314.2	40.5	39.5

Means with different letters (Statistical significant) are significantly different at $p < 0.05$ using Tukey's method.

C. Shoot K^+/Na^+ ratio (n=3, biological replications)

Plants	Species	Average shoot K^+/Na^+ ratio								7d/0d ratio	SE			
		0d	Statistical significance	1d	Statistical significance	3d	Statistical significance	7d	Statistical significance		0d	1d	3d	7d
Rice	*O. sativa*	12.52	abc	0.56	ij	0.18	j	0.16	j	0.012	3.80	0.01	0.01	0.01
OKN4	*S. humilis*	4.16	fghij	3.01	ghij	2.14	hij	1.33	ij	0.320	0.31	0.50	0.25	0.12
OKN5	*P. aquaticum*	8.21	bcdefg	0.88	ij	0.81	ij	1.18	ij	0.144	3.57	0.06	0.06	0.23
OKN10	*C. dactylon*	14.51	a	4.04	fghij	1.85	hij	0.83	ij	0.057	4.57	0.83	0.47	0.04
OKN11	*Unknown*	5.45	defghij	4.52	efghij	3.43	fghij	1.99	hij	0.364	2.78	0.18	0.77	0.15
OKN12	*S. Virginicus*	4.26	fghij	2.88	ghij	1.91	hij	1.71	ij	0.401	0.48	0.61	0.38	0.10
NGT2	*C. dactylon*	12.91	ab	3.95	fghij	3.30	fghij	3.13	ghij	0.243	4.39	1.05	0.86	0.67
NGT3	*C. dactylon*	10.88	abcd	8.80	bcdef	3.35	fghij	3.01	ghij	0.277	2.20	1.42	0.29	0.07
CHB1	*C. dactylon*	7.29	cdefgh	5.14	efghij	3.36	fghij	2.91	ghij	0.399	2.53	0.63	0.19	0.75
SZO10	*C. dactylon*	5.98	defghi	3.89	fghij	3.79	fghij	2.45	hij	0.410	0.78	0.24	0.33	0.08
HYG2	*C. dactylon*	10.16	abcde	2.81	ghij	2.14	hij	1.90	hij	0.187	4.49	1.36	0.15	0.09

Means with different letters (Statistical significant) are significantly different at $p < 0.05$ using Tukey's method.

D. Root Na^+ content (n=3, biological replications)

Plants	Species	Average root Na^+ content (μmol g^{-1} DW)								7d/0d ratio	SE			
		0d	Statistical significance	1d	Statistical significance	3d	Statistical significance	7d	Statistical significance		0d	1d	3d	7d
Rice	*O. sativa*	468.3	fg	2448.4	bcde	2592.4	bcd	3467.4	ab	7.4	47.6	321.2	382.4	639.1
OKN4	*S. humilis*	271.0	g	321.8	fg	407.6	fg	1498.4	cdefg	5.5	166.1	141.7	176.1	256.9
OKN5	*P. aquaticum*	155.9	g	530.7	efg	1009.4	defg	1727.8	bcdefg	11.1	83.1	202.0	190.9	792.1
OKN10	*C. dactylon*	97.3	g	598.6	efg	499.2	fg	1876.8	bcdefg	19.3	60.6	38.8	276.8	120.2
OKN11	*Unknown*	169.8	g	552.6	efg	915.9	defg	1551.6	bcdefg	9.1	25.6	87.6	100.8	500.1
OKN12	*S. Virginicus*	48.2	g	426.9	defg	1781.0	bcdefg	619.8	efg	12.9	29.1	14.3	1376.6	76.9
NGT2	*C. dactylon*	125.1	g	351.6	fg	1739.7	bcdefg	1007.7	defg	8.1	54.0	274.6	257.7	461.6
NGT3	*C. dactylon*	186.8	g	1013.3	defg	722.6	defg	765.9	defg	4.1	58.4	540.6	29.7	145.7
CHB1	*C. dactylon*	256.0	g	468.1	fg	1760.3	bcdefg	4780.6	a	18.7	95.0	13.4	363.3	2552.0
SZO10	*C. dactylon*	56.4	g	451.2	fg	822.4	defg	2218.2	bcdef	39.3	24.1	170.5	189.0	804.6
HYG2	*C. dactylon*	195.1	g	1253.5	cdefg	3186.5	abc	1487.1	cdefg	7.6	20.5	801.2	1151.5	397.8

Means with different letters (Statistical significant) are significantly different at $p < 0.05$ using Tukey's method.

E. Root K^+ content (n=3, biological replications)

Plants	Species	Average root K^+ content (µmol g^{-1} DW)								7d/0d ratio	SE			
		0d	Statistical significance	1d	Statistical significance	3d	Statistical significance	7d	Statistical significance		0d	1d	3d	7d
Rice	*O. sativa*	2570.0	ab	680.4	cde	376.3	cde	493.8	cde	0.19	932.3	47.8	73.0	108.5
OKN4	*S. humilis*	294.1	cde	226.4	de	320.6	cde	796.9	bcde	2.71	231.6	106.6	145.8	156.0
OKN5	*P. aquaticum*	1156.1	bcde	403.4	cde	458.5	cde	446.0	cde	0.39	353.1	83.4	105.9	237.7
OKN10	*C. dactylon*	372.6	cde	319.2	cde	150.4	de	331.2	cde	0.89	203.4	40.2	76.9	19.3
OKN11	*Unknown*	967.9	bcde	263.5	cde	503.8	cde	1108.9	bcde	1.15	332.5	35.9	52.9	570.8
OKN12	*S. Virginicus*	338.1	cde	853.3	cde	1254.9	bcde	347.5	cde	1.03	196.6	54.8	961.4	59.7
NGT2	*C. dactylon*	968.4	e	601.8	cde	906.7	ab	363.9	bcde	0.38	169.9	127.3	30.7	66.4
NGT3	*C. dactylon*	895.5	bcde	897.1	bcde	431.1	cde	390.6	cde	0.44	142.5	473.5	40.5	57.9
CHB1	*C. dactylon*	569.7	cde	181.2	de	433.3	cde	738.2	bcde	1.30	209.1	11.6	204.4	342.3
SZO10	*C. dactylon*	286.0	cde	227.5	de	203.1	de	411.2	cde	1.44	144.2	92.5	53.9	219.5
HYG2	*C. dactylon*	447.8	bcde	160.8	cde	602.9	bcde	219.1	cde	0.49	88.1	396.7	423.2	76.9

Means with different letters (Statistical significant) are significantly different at $p < 0.05$ using Tukey's method.

F. Root K^+/Na^+ ratio (n=3, biological replications)

Plants	Species	Average root K^+/Na^+ ratio								7d/0d ratio	SE			
		0d	Statistical significance	1d	Statistical significance	3d	Statistical significance	7d	Statistical significance		0d	1d	3d	7d
Rice	*O. sativa*	3.50	cdefg	0.285	i	0.144	i	0.144	i	0.041	0.16	0.029	0.012	0.019
OKN4	*S. humilis*	1.74	efghi	0.639	hi	0.740	hi	0.529	hi	0.303	1.07	0.121	0.059	0.051
OKN5	*P. aquaticum*	6.27	ab	1.472	ghi	0.458	hi	0.243	i	0.039	0.05	1.000	0.077	0.035
OKN10	*C. dactylon*	3.81	cde	0.529	hi	0.320	i	0.179	i	0.047	0.78	0.032	0.018	0.021
OKN11	*Unknown*	6.42	a	0.520	hi	0.566	hi	0.618	hi	0.096	3.08	0.154	0.094	0.215
OKN12	*S. Virginicus*	2.13	efghi	0.776	hi	0.717	hi	0.555	hi	0.260	0.35	0.100	0.009	0.030
NGT2	*C. dactylon*	3.80	cdef	0.467	hi	0.361	i	0.261	i	0.069	0.36	0.065	0.048	0.049
NGT3	*C. dactylon*	5.53	abc	0.890	hi	0.594	hi	0.522	hi	0.094	1.27	0.047	0.035	0.036
CHB1	*C. dactylon*	2.52	defgh	0.387	i	0.227	i	0.165	i	0.065	0.55	0.023	0.068	0.014
SZO10	*C. dactylon*	4.29	bcd	0.520	hi	0.243	i	0.167	i	0.039	1.07	0.059	0.008	0.031
HYG2	*C. dactylon*	5.13	abc	0.484	hi	0.283	i	0.252	i	0.049	0.89	0.022	0.081	0.024

Means with different letters (Statistical significant) are significantly different at $p < 0.05$ using Tukey's method.

Supplementary Table S3: Changes in proline content in shoot and root of turfgrasses and rice under 300 mM NaCl condition

A. Shoot proline content (n=3, biological replications)

Plants	Species	Average shoot proline content (μmol g^{-1} FW)				7d/0d ratio	SE	
		0d	Statistical significance	7d	Statistical significance		0d	7d
Rice	*O. sativa*	1.8	f	15.7	f	8.9	0.1	1.6
OKN5	*S. humilis*	3.0	f	78.7	de	25.9	0.6	48.5
OKN11	*P. aquaticum*	1.2	f	25.8	ef	21.2	0.6	14.6
NGT2	*C. dactylon*	2.5	f	176.0	bc	70.4	0.4	14.0
CHB1	*Unknown*	3.1	f	209.3	b	67.7	1.2	6.6
OKN4	*S. Virginicus*	3.2	f	87.0	d	27.1	0.2	0.8
OKN10	*C. dactylon*	1.0	f	256.1	a	250.6	0.5	20.0
OKN12	*C. dactylon*	2.7	f	143.3	c	52.9	0.2	21.2
NGT3	*C. dactylon*	2.0	f	179.4	bc	88.6	0.6	9.5
SZO10	*C. dactylon*	1.4	f	273.3	a	189.2	0.2	16.9
HYG2	*C. dactylon*	1.7	f	156.0	bc	89.2	0.1	6.0

Means with different letters (Statistical significant) are significantly different at $p < 0.05$ using Tukey’s method.

B. Root proline content (n=3, biological replications)

Plants	Species	Average root proline content (μmol g^{-1} FW)				7d/0d ratio	SE	
		0d	Statistical significance	7d	Statistical significance		0d	7d
Rice	*O. sativa*	2.6	f	4.0	f	1.5	0.6	0.3
OKN5	*S. humilis*	2.5	f	2.2	f	0.9	0.1	1.1
OKN11	*P. aquaticum*	1.2	f	6.7	f	5.6	0.5	1.0
NGT2	*C. dactylon*	2.3	f	69.8	e	30.7	0.1	6.0
CHB1	*Unknown*	3.9	f	96.3	bcd	24.5	0.7	12.1
OKN4	*S. Virginicus*	5.9	f	83.7	cde	14.1	0.7	15.7
OKN10	*C. dactylon*	1.6	f	107.6	b	67.8	0.2	9.9
OKN12	*C. dactylon*	3.2	f	90.1	bcd	28.0	0.6	8.6
NGT3	*C. dactylon*	0.8	f	127.7	a	155.6	0.3	5.2
SZO10	*C. dactylon*	1.6	f	100.0	bc	64.4	0.1	7.1
HYG2	*C. dactylon*	1.6	f	79.9	de	49.3	0.2	5.0

Means with different letters (Statistical significant) are significantly different at $p < 0.05$ using Tukey’s method.
